# Supplementary material for: NMR-Based Metabolomics to Analyze the Effects of a Series of Monoamine Oxidases-B Inhibitors on U251 Cells
Source: Biomolecules. 2023 Mar 27;13(4):600. doi: 10.3390/biom13040600 (PMC10136171; doi:10.3390/biom13040600)
Supplement: Supplementary file 1 [file biomolecules-13-00600-s001.zip › biomolecules-2167680-supplementary.pdf]

*Supplemental Materials*

# NMR-Based Metabolomics to Analyze the Effects of a Series of Monoamine Oxidases-B Inhibitors on U251 Cells

Zili Guo, Jinping Gu, Miao Zhang, Feng Su, Weike Su, Yuanyuan Xie

**7 Supplementary Figures**

**5 Supplementary Tables**

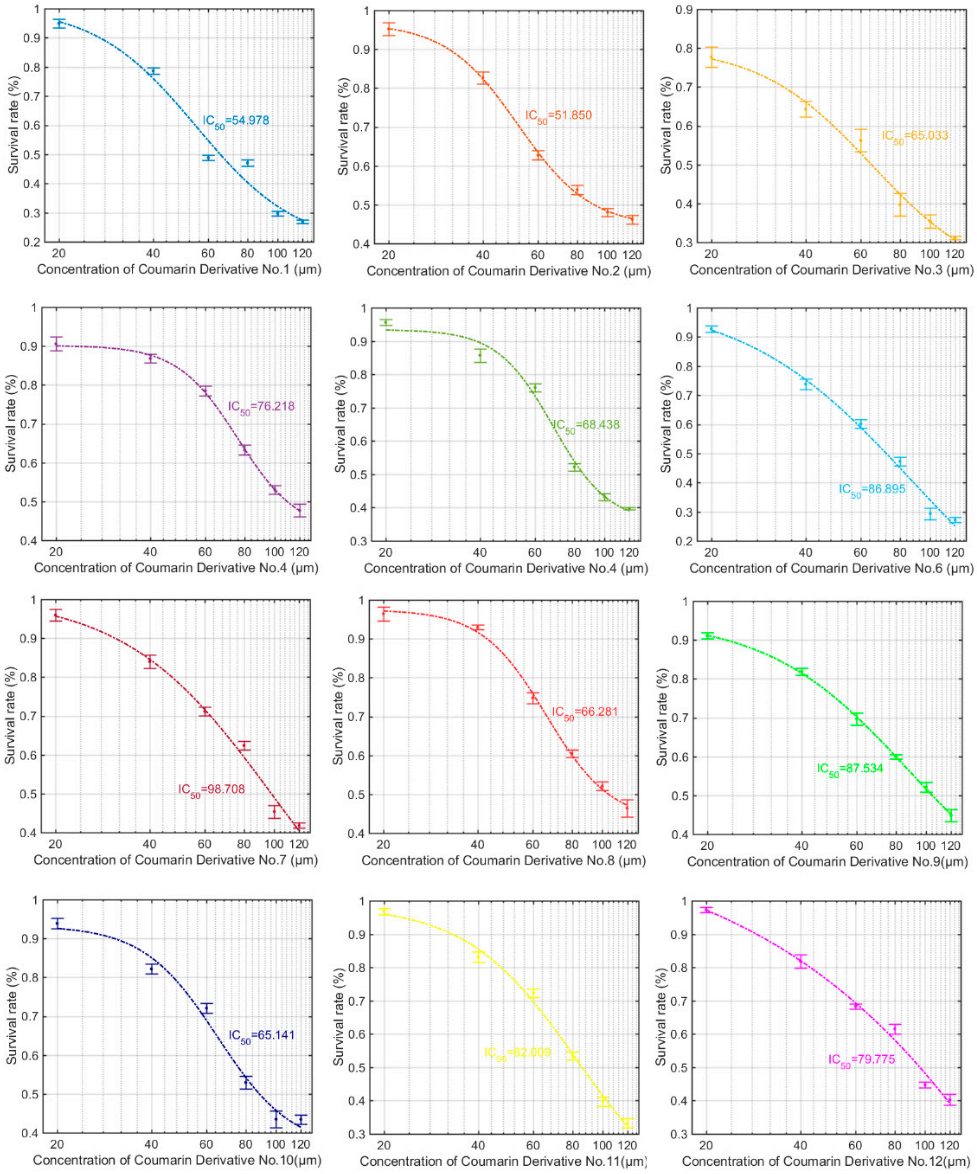


**Figure S1.** The $IC_{50}$ value of coumarin derivatives in the U251 cells.

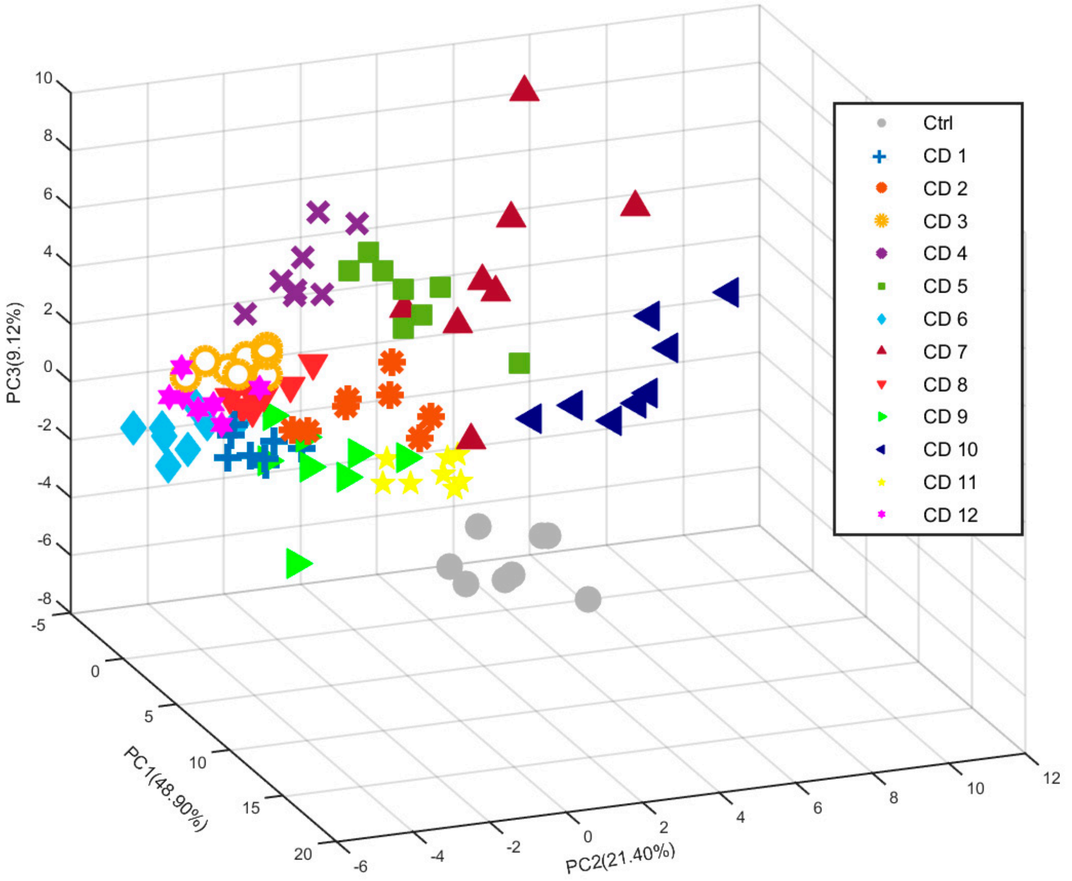


**Figure S2.** PCA scores plots of relative concentration of metabolites data from twelve coumarin derivatives and control group.

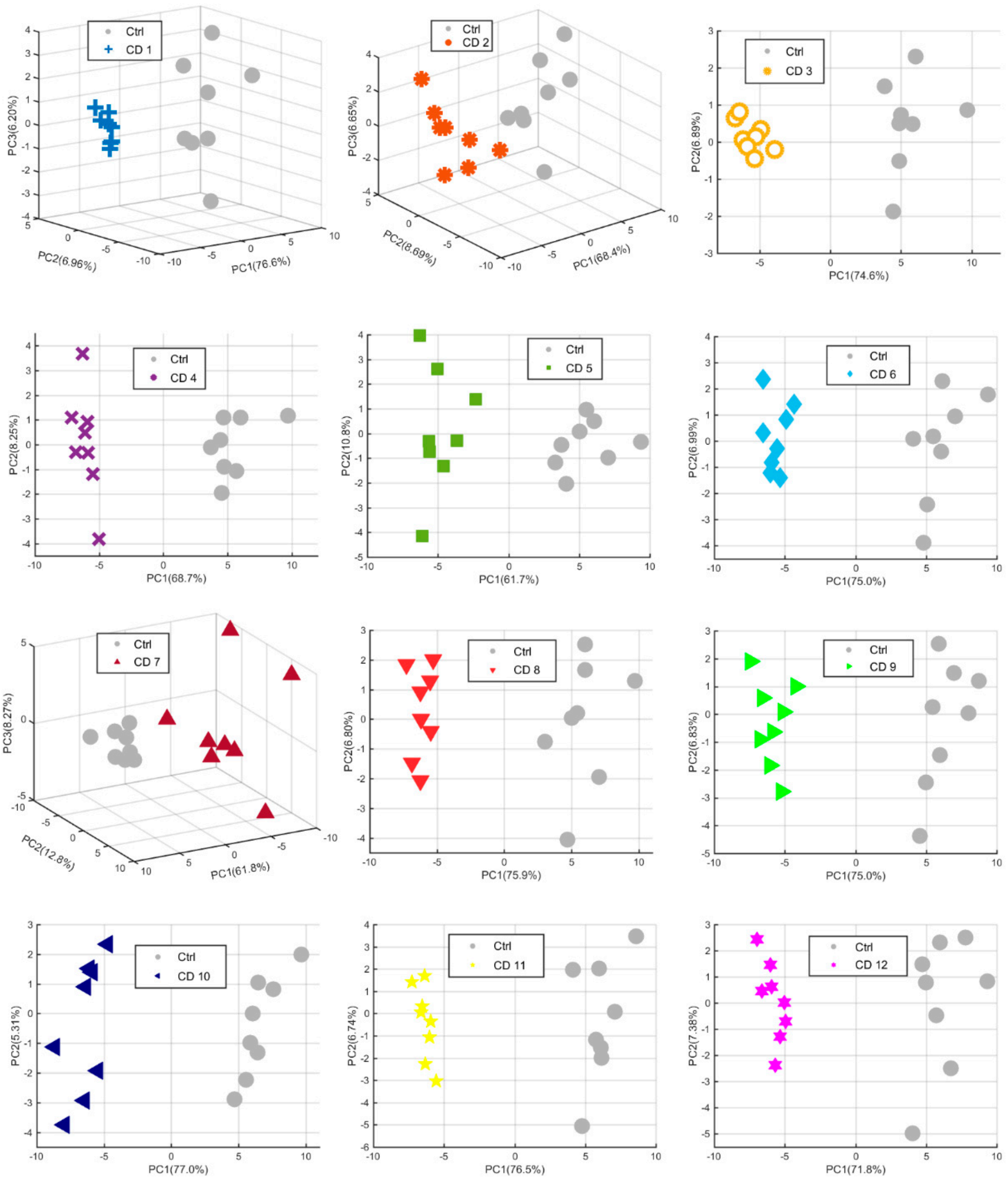


**Figure S3.** PCA scores plots of relative concentration of metabolites data from each coumarin derivative compared with control group.

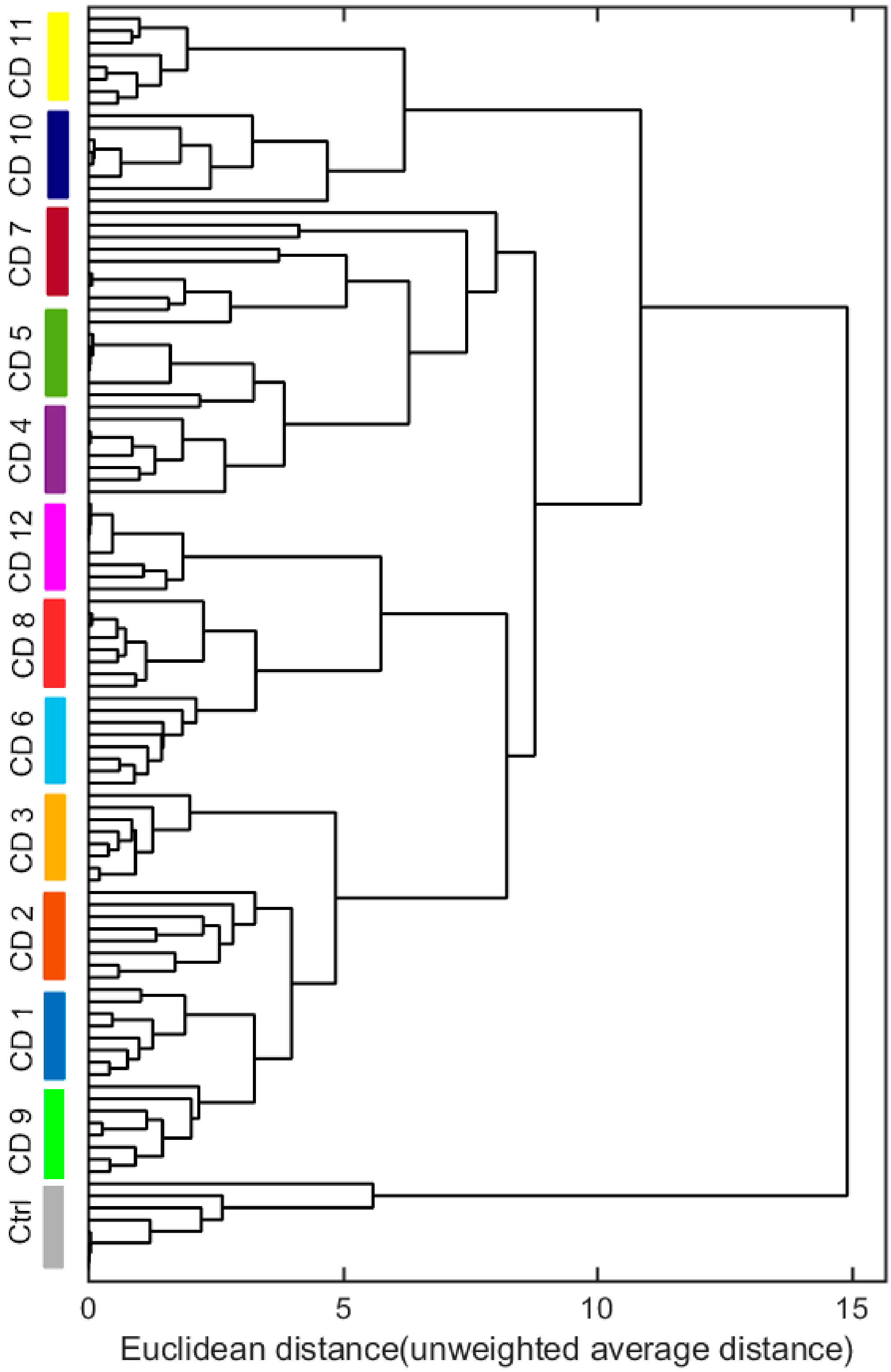


**Figure S4.** Result of HCA of relative concentration of metabolites data from twelve coumarin derivatives and control group.

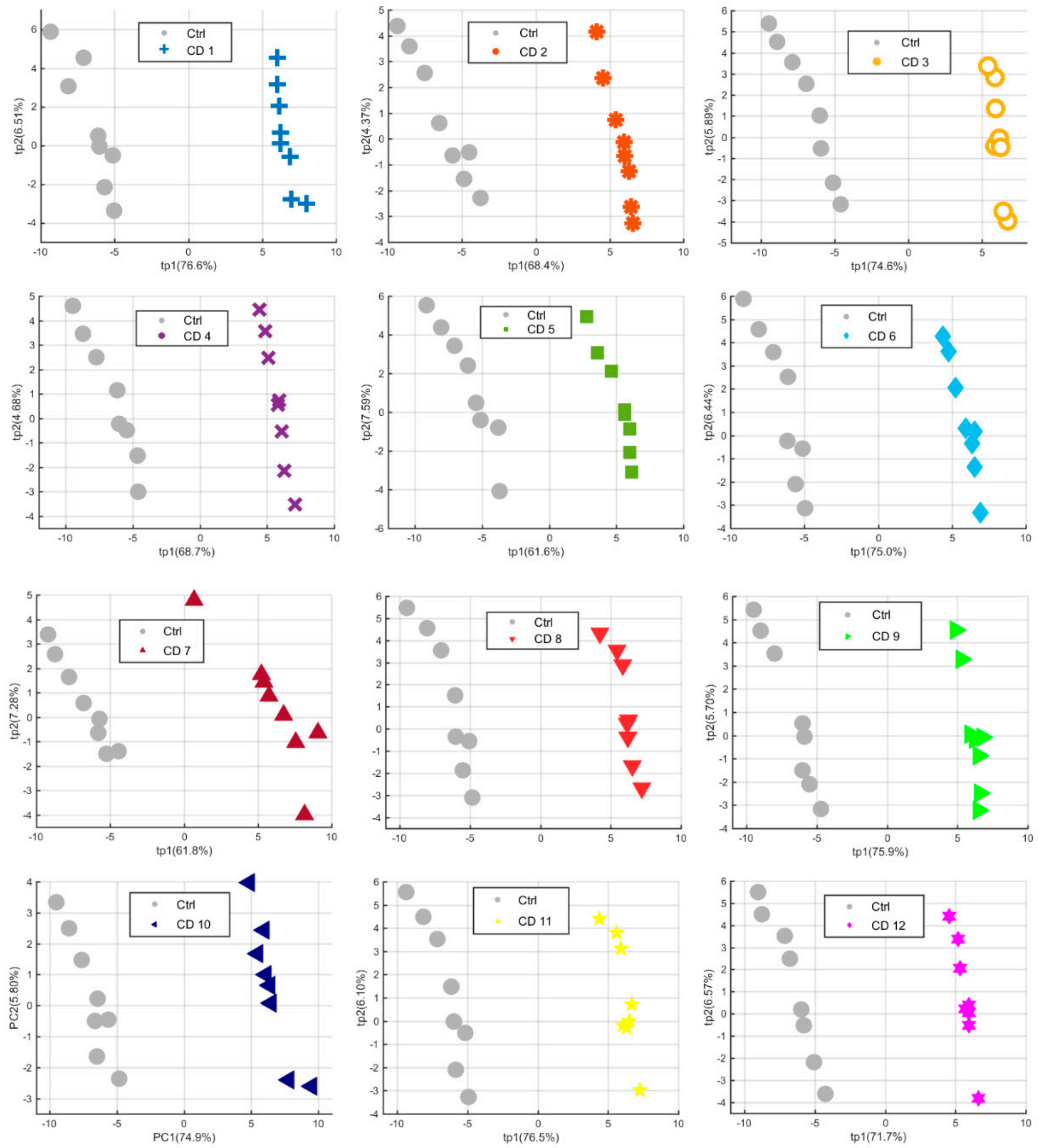


**Figure S5.** PLS-DA scores plot of relative concentration of metabolites data from each coumarin derivative compared with control group.

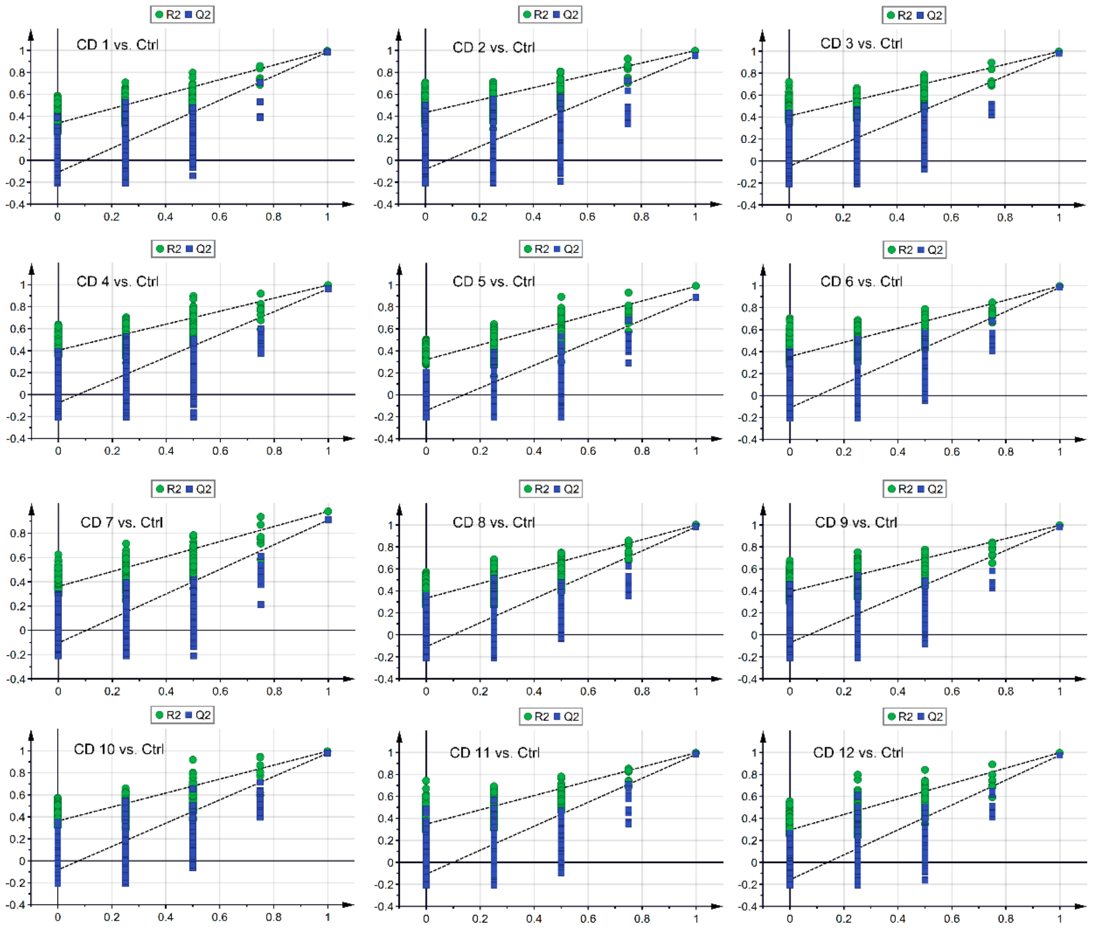


**Figure S6.** Corresponding permutation tests of PLS-DA models of relative concentration of metabolites data from each coumarin derivative compared with control group.

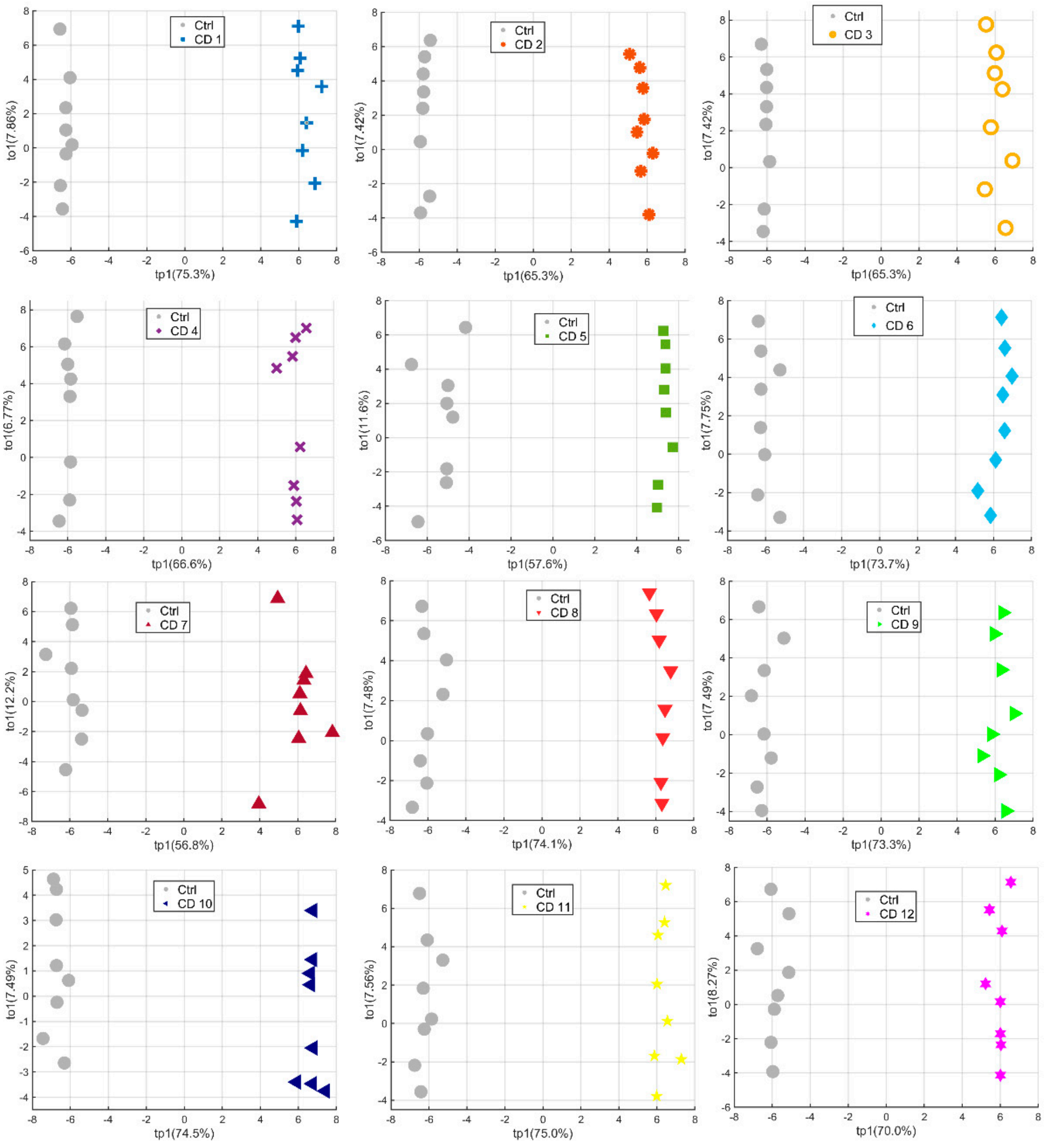


**Figure S7. O**PLS-DA scores plot of relative concentration of metabolites data from each coumarin derivative compared with control group.

**Table S1.** The $IC_{50}$ value of coumarin derivatives in the U251 cells

| No. | Compound structure | $IC_{50}$ / μm |
|---|---|---|
| CD1 | 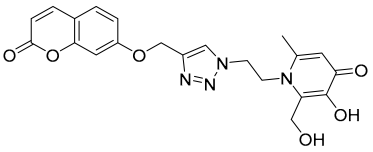 | 54.978 |
| CD2 | 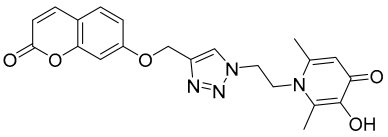 | 51.850 |
| CD3 | 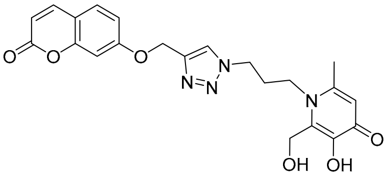 | 65.033 |
| CD4 | 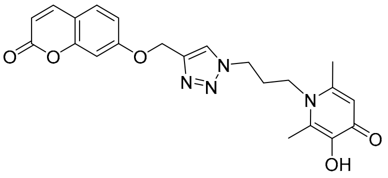 | 76.218 |
| CD5 | 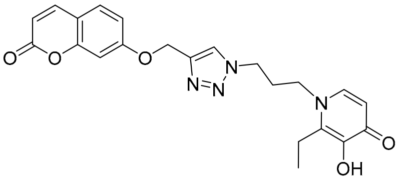 | 68.438 |
| CD6 | 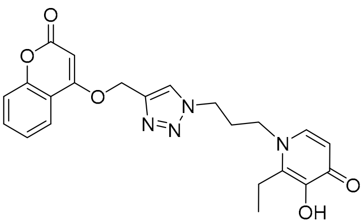 | 86.896 |
| CD7 | 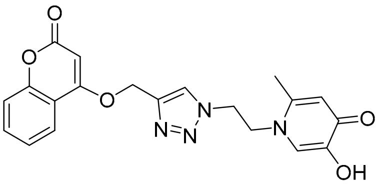 | 98.708 |

| | | |
|---|---|---|
| CD8 | 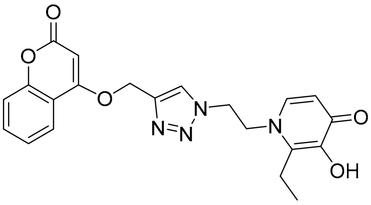 | 66.281 |
| CD9 | 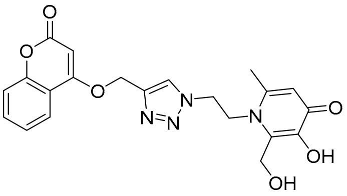 | 87.534 |
| CD10 | 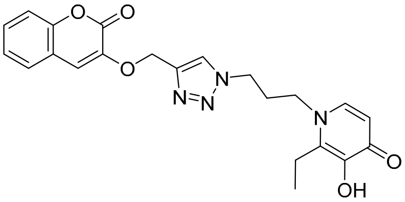 | 65.141 |
| CD11 | 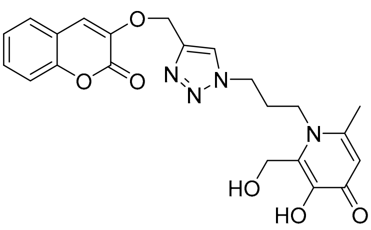 | 82.009 |
| CD12 | 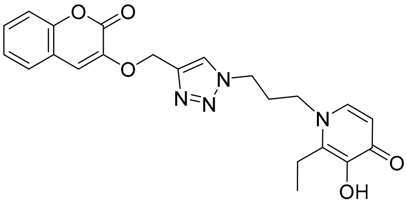 | 79.775 |

**Table S2.** The detailed information of twelve coumarin derivatives in our work.

| No. | Compound structure | Data Representation |
| --- | --- | --- |
| CD1 | 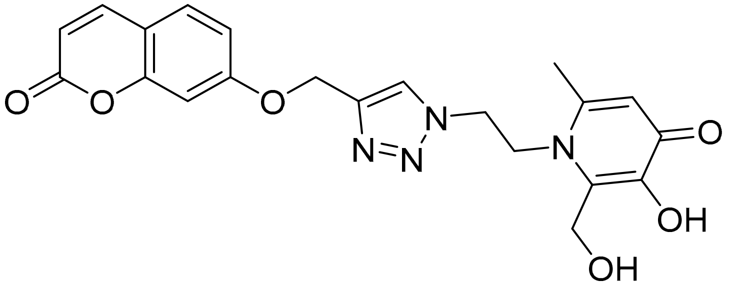 | **3-hydroxy-2-(hydroxymethyl)-6-methyl-1-(2-(4-(((2-oxo-2H-chromen-7-yl)oxy)methyl)-1H-1,2,3-triazol-1-yl)ethyl)pyridin-4(*1H*)-one:** yellow solid, yield: 81%; m.p.: 212-215 ºC; $^{1}$H NMR (600 MHz, DMSO-$d_6$) δ 8.32 (s, 1H), 8.02 (d, $J$ = 9.5 Hz, 1H), 7.66 (d, $J$ = 8.7 Hz, 1H), 7.15 (d, $J$ = 2.5 Hz, 1H), 7.10 (s, 1H), 7.02 (dd, $J$ = 8.6, 2.4 Hz, 1H), 6.32 (d, $J$ = 9.5 Hz, 1H), 5.30 (s, 2H), 4.98 (d, $J$ = 5.9 Hz, 2H), 4.95 (d, $J$ = 5.9 Hz, 2H), 4.60 (s, 2H), 2.42 (s, 3H). $^{13}$C NMR (151 MHz, DMSO-$d_6$) δ 161.4, 160.7, 160.6, 155.7, 149.6, 144.7, 142.9, 142.8, 142.6, 130.0, 126.3, 113.5, 113.3, 113.2, 113.1, 102.1, 61.9, 53.1, 50.5, 48.4, 20.1. HRMS (ESI) calcd for $C_{21}H_{21}N_4O_6$ [M+H]$^+$: 425.1456; found: 425.1439. |
| CD2 | 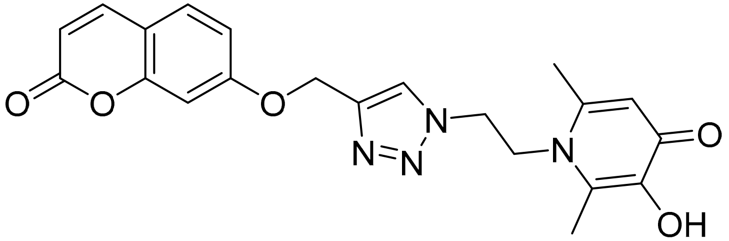 | **3-hydroxy-2,6-dimethyl-1-(2-(4-(((2-oxo-2H-chromen-7-yl)oxy)methyl)-1H-1,2,3-triazol-1-yl)ethyl)pyridin-4(*1H*)-one:** white solid, yield: 93%; m.p.: 256-249 ºC; $^{1}$H NMR (600 MHz, DMSO-$d_6$) δ 8.34 (s, 1H), 8.02 (d, $J$ = 9.5 Hz, 1H), 7.66 (d, $J$ = 8.6 Hz, 1H), 7.14 (s, 1H), 7.03 (s, 1H), 7.01 (dd, $J$ = 8.6, 2.5 Hz, 1H), 6.32 (d, $J$ = 9.5 Hz, 1H), 5.30 (s, 2H), 4.93 (t, $J$ = 6.3 Hz, 2H), 4.80 (t, $J$ = 6.2 Hz, 2H), 2.41 (s, 3H), 2.34 (s, 3H). $^{13}$C NMR (151 MHz, DMSO-$d_6$) δ 161.4, 160.7, 159.1, |

| | | |
|---|---|---|
| | | 155.7, 148.4, 144.8, 143.1, 142.9, 141.9, 130.0, 126.4, 113.4, 113.2, 113.1, 112.6, 102.1, 61.9, 51.1, 47.7, 20.3, 13.6. HRMS (ESI) calcd for $C_{21}H_{21}N_4O_5$ [M+H]$^+$: 409.1518; found: 409.1506. |
| CD3 | 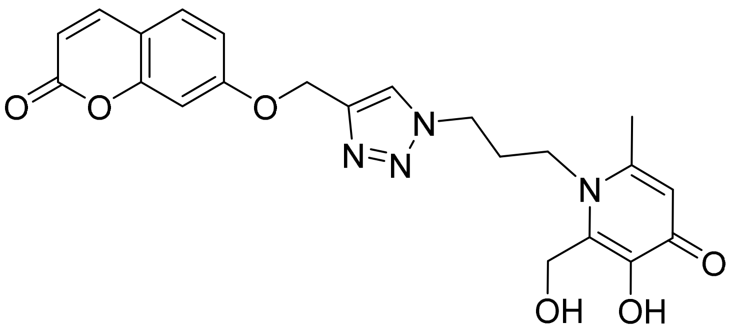 | **3-hydroxy-2-(hydroxymethyl)-6-methyl-1-(3-(4-(((2-oxo-2H-chromen-7-yl)oxy)methyl)-1H-1,2,3-triazol-1-yl)propyl)pyridin-4(*1H*)-one:** yellow solid, yield: 84%; m.p.: 189-192 ºC; $^1$H NMR (600 MHz, DMSO-$d_6$) δ 8.41 (s, 1H), 8.02 (d, *J* = 9.5 Hz, 1H), 7.66 (d, *J* = 8.6 Hz, 1H), 7.15 (s, 1H), 7.12 (s, 1H), 7.02 (d, *J* = 8.5 Hz, 1H), 6.31 (dd, *J* = 9.4, 1.7 Hz, 1H), 5.29 (s, 2H), 4.76 (s, 2H), 4.61 (t, *J* = 6.7 Hz, 2H), 4.45 (t, *J* = 8.5 Hz, 2H), 2.54 (s, 3H), 2.40 (dq, *J* = 13.3, 6.9 Hz, 2H). $^{13}$C NMR (151 MHz, DMSO-$d_6$) δ 163.5, 161.5, 160.7, 160.0, 155.7, 148.8, 144.7, 142.7, 142.5, 142.3, 130.0, 125.5, 113.5, 113.3, 113.1, 102.0. HRMS (ESI) calcd for $C_{22}H_{23}N_4O_6$ [M+H]$^+$: 439.1612; found: 439.1606. |
| CD4 | 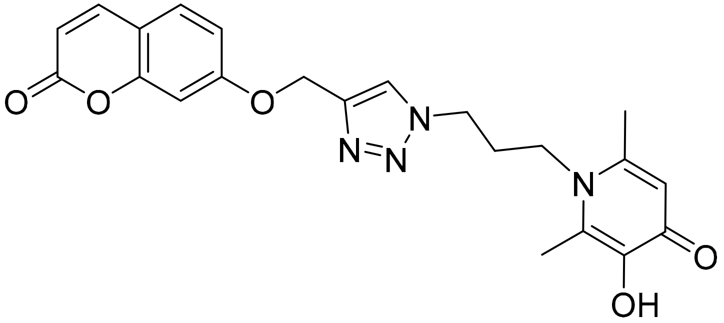 | **3-hydroxy-2,6-dimethyl-1-(3-(4-(((2-oxo-2H-chromen-7-yl)oxy)methyl)-1H-1,2,3-triazol-1-yl)propyl)pyridin-4(*1H*)-one:** white solid, yield: 87%; m.p.: 263-265 ºC; $^1$H NMR (600 MHz, DMSO-$d_6$) δ 8.43 (s, 1H), 8.01 (d, *J* = 9.4 Hz, 1H), 7.66 (d, *J* = 8.5 Hz, 1H), 7.15 (s, 1H), 7.06 (s, 1H), 7.02 (d, *J* = 8.7 Hz, 1H), 6.30 (d, *J* = 9.4 Hz, 1H), 5.30 (s, 2H), 4.62 (t, *J* = 6.7 Hz, 2H), 4.34-4.18 (m, 2H), 2.51 (d, *J* = 7.0 |

| | | |
|---|---|---|
| | | Hz, 5H), 2.47 (s, 3H). $^{13}$C NMR (151 MHz, DMSO-$d_6$) δ 161.5, 160.7, 158.6, 155.7, 147.6, 144.7, 142.6, 142.5, 141.8, 130.0, 125.6, 113.4, 113.1, 113.1, 112.6, 102.1, 62.2, 49.1, 47.1, 28.8, 20.3, 13.4. HRMS (ESI) calcd for $C_{22}H_{23}N_4O_5$ [M+H]$^+$: 423.1663; found: 423.1676. |
| CD5 | 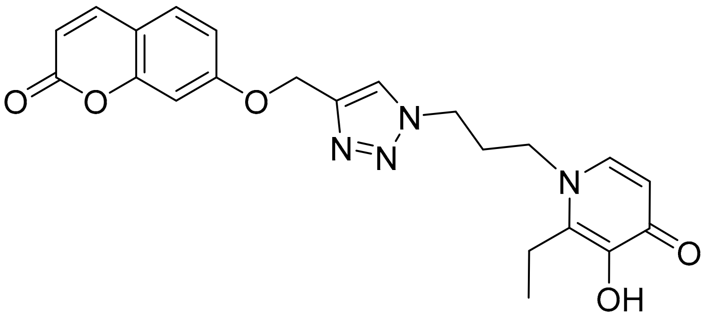 | **2-ethyl-3-hydroxy-1-(3-(4-(((2-oxo-2H-chromen-7-yl)oxy)methyl)-1H-1,2,3-triazol-1-yl)propyl)pyridin-4(*1H*)-one:** white solid, yield: 91%; m.p.: 204-206 ºC; $^1$H NMR (600 MHz, DMSO-$d_6$) δ 8.29 (s, 1H), 8.02 (d, *J* = 9.5 Hz, 1H), 7.88 (d, *J* = 7.1 Hz, 1H), 7.66 (d, *J* = 8.6 Hz, 1H), 7.13 (d, *J* = 2.4 Hz, 1H), 7.05 (d, *J* = 6.6 Hz, 1H), 7.00 (dd, *J* = 8.6, 2.4 Hz, 1H), 6.32 (d, *J* = 9.5 Hz, 1H), 5.27 (s, 2H), 4.92 (dd, *J* = 6.8, 4.5 Hz, 2H), 4.86 (d, *J* = 5.8 Hz, 2H), 2.69 (q, *J* = 7.4 Hz, 2H), 1.09 (t, *J* = 7.5 Hz, 3H). $^{13}$C NMR (151 MHz, DMSO-$d_6$) δ 161.4, 160.7, 159.5, 155.7, 146.9, 144.7, 143.2, 142.8, 138.8, 130.2, 126.7, 113.3, 113.2, 113.1, 111.3, 102.1, 61.9, 55.1, 49.8, 19.8, 12.1. HRMS (ESI) calcd for $C_{21}H_{21}N_4O_5$ [M+H]$^+$: 409.1506; found: 409.1512. |

| | | |
|---|---|---|
| CD6 | 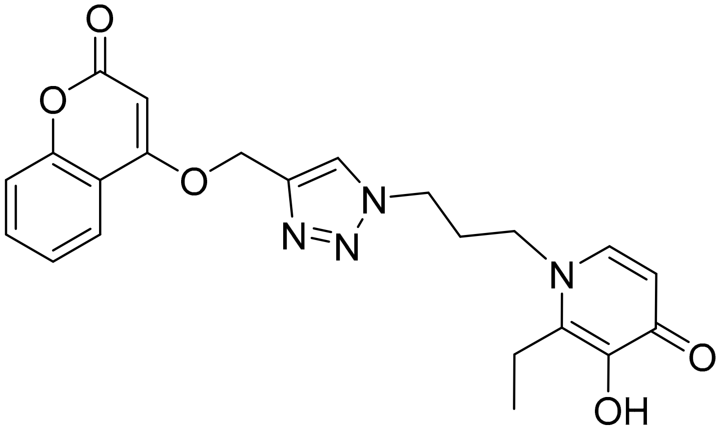 | **2-ethyl-3-hydroxy-1-(3-(4-(((2-oxo-2H-chromen-4-yl)oxy)methyl)-1H-1,2,3-triazol-1-yl)propyl)pyridin-4(*1H*)-one:** white solid, yield: 90%; m.p.: 192-195 ºC; $^{1}$H NMR (600 MHz, DMSO-$d_6$) δ 8.31 (s, 1H), 8.12 (d, *J* = 7.0 Hz, 1H), 7.65 (d, *J* = 7.8 Hz, 1H), 7.46 (d, *J* = 8.0 Hz, 1H), 7.38 (d, *J* = 8.4 Hz, 1H), 7.33 (t, *J* = 7.2 Hz, 1H), 7.18 (d, *J* = 7.2 Hz, 1H), 6.21 (s, 1H), 5.38 (s, 2H), 4.51 (t, *J* = 6.6 Hz, 2H), 4.41-4.37 (m, 2H), 2.87 (q, *J* = 7.5 Hz, 2H), 2.32–2.28 (m, 2H), 1.22 (t, *J* = 7.2 Hz, 3H). $^{13}$C NMR (150 MHz, DMSO-$d_6$) δ 163.6 , 161.5, 158.3, 152.8, 145.3, 141.7, 140.1, 138.5, 131.2, 126.7, 123.4, 125.1, 116.1, 114.6, 112.7, 92.7, 64.6, 51.2, 46.4, 30.8, 20.2, 12.5. HRMS (ESI) calcd for $C_{22}H_{23}N_4O_5$ $[M+H]^+$: 423.1815; found: 423.1826. |
| CD7 | 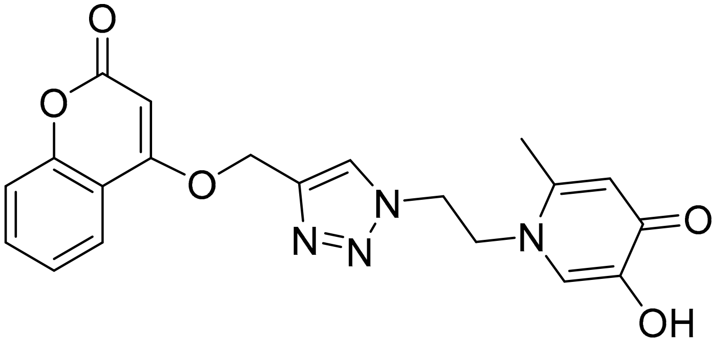 | **5-hydroxy-2-methyl-1-(2-(4-(((2-oxo-2H-chromen-4-yl)oxy)methyl)-1H-1,2,3-triazol-1-yl)ethyl)pyridin-4(*1H*)-one:** white solid, yield: 86%; m.p.: 235-237ºC; $^{1}$H NMR (600 MHz, DMSO-$d_6$) *δ* 8.30 (s, 1H), 8.01 (d, *J* = 9.6 Hz, 1H), 7.88 (d, *J* = 7.2 Hz, 1H), 7.65 (d, *J* = 8.4 Hz, 1H), 7.12 (d, *J* = 1.6 Hz, 1H), 7.05 (d, *J* = 7.2 Hz, 1H), 6.99 (dd, *J* = 2.0, 8.4 Hz, 1H), 6.30 (d, *J* = 9.2 Hz, 1H), 5.26 (s, 2H), 4.92 (d, *J* = 6.0 Hz, 4H), 2.40 (s, 3H); $^{13}$C NMR (150 MHz, DMSO-$d_6$) δ 164.7, 161.9, 160.5, 153.2, 148.0, 144.2, 141.6, 133.3, 131.7, 125.7, 124.7, 123.3, 116.9, 115.5, |

| | | |
|---|---|---|
| | | 114.5, 91.8, 63.2, 53.1, 47.0, 30.1, 18.8. HRMS (ESI) calcd for $C_{20}H_{19}N_4O_5$ $[M+H]^+$: 395.1315; found: 395.1321. |
| CD8 | 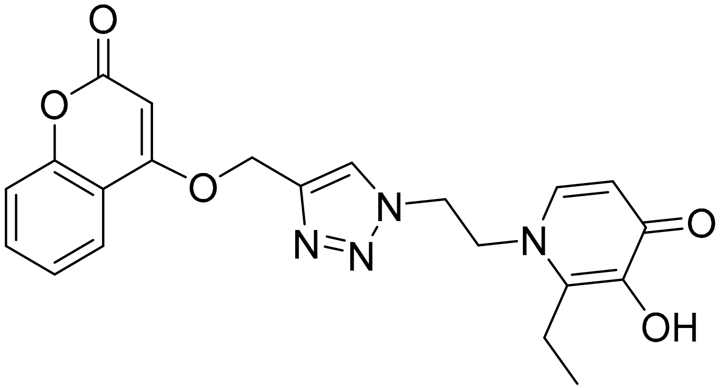 | **2-ethyl-3-hydroxy-1-(2-(4-(((2-oxo-2H-chromen-4-yl)oxy)methyl)-1H-1,2,3-triazol-1-yl)ethyl)pyridin-4(*1H*)-one:**;white solid, yield: 90%; m.p.: 253-256 ºC $^1$H NMR (600 MHz, DMSO-$d_6$) δ 8.31 (s, 1H), 8.13 (d, *J* = 7.0 Hz, 1H), 7.81 (d, *J* = 7.8 Hz, 1H), 7.63 (dd, *J* = 8.4, 7.2, Hz, 1H), 7.41 (d, *J* = 8.4 Hz, 1H), 7.31 (t, *J* = 7.2 Hz, 1H), 7.18 (d, *J* = 7.2 Hz, 1H), 6.18 (s, 1H), 5.45 (s, 2H), 4.58 (t, *J* = 6.9 Hz, 2H), 2.87 (q, *J* = 7.5 Hz, 2H), 2.44 (t, *J* = 6.6 Hz, 2H), 1.11 (t, *J* = 7.5 Hz, 3H). $^{13}$C NMR (150 MHz, DMSO-$d_6$) δ 167.1, 163.0, 159.3, 152.2, 145.6, 143.8, 140.7, 138.2, 133.7, 126.1, 124.3, 123.3, 116.7, 111.6, 110.7, 91.3, 62.3, 51.3, 47.3, 32.1, 12.6. HRMS (ESI) calcd for $C_{21}H_{21}N_4O_5$ $[M+H]^+$: 409.1506; found: 409.1500. |

| | | |
|---|---|---|
| CD9 | 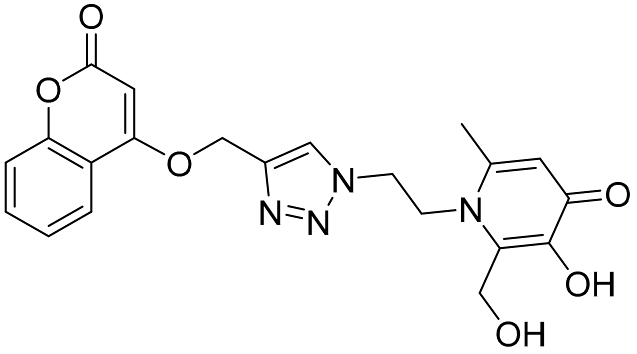 | **3-hydroxy-2-(hydroxymethyl)-6-methyl-1-(2-(4-(((2-oxo-2H-chromen-4-yl)oxy)methyl)-1H-1,2,3-triazol-1-yl)ethyl)pyridin-4(*1H*)-one:** white solid, yield: 89%; m.p.: 142-148 ºC; $^{1}$H NMR (600 MHz, DMSO-$d_6$) δ 8.35 (s, 1H), 8.07 (d, *J* = 8.4 Hz, 1H), 7.71 (d, *J* = 8.4 Hz, 1H), 7.35 (d, *J* = 6.6 Hz, 1H), 7.26 (s, 1H), 7.13 (d, *J* = 8.4Hz, 1H), 6.21 (d, *J* = 8.4 Hz, 1H), 5.43 (s, 2H), 4.95 (d, *J* = 6.0 Hz, 2H), 4.72 (d, *J* = 6.0 Hz, 2H), 4.60 (s, 2H), 2.36 (s, 3H). $^{13}$C NMR (150 MHz, DMSO-$d_6$) δ 165.3 , 162.1 , 161.4 , 152.7, 141.7, 144.5, 143.1, 142.7, 142.3, 131.1, 128.6, 111.4, 113.3, 112.0, 113.1, 101.5, 63.7, 52.9, 50.3, 47.4, 20.5. HRMS (ESI) calcd for $C_{21}H_{21}N_4O_6$ [M+H]$^+$: 425.1415; found: 425.1418. |
| CD10 | 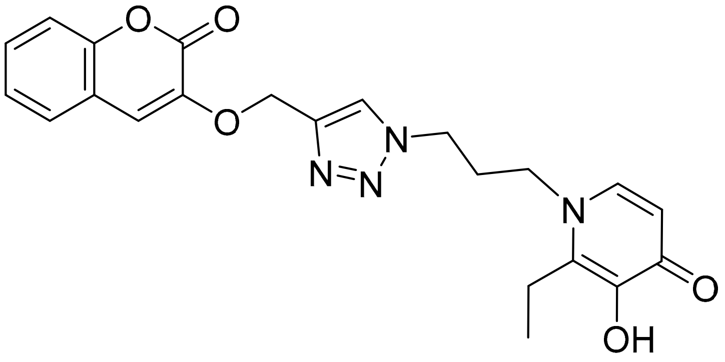 | **3-hydroxy-2-(hydroxymethyl)-6-methyl-1-(2-(4-(((2-oxo-2H-chromen-3-yl)oxy)methyl)-1H-1,2,3-triazol-1-yl)ethyl)pyridin-4(*1H*)-one:** white solid, yield: 85%; m.p.: 168-170 ºC; $^{1}$H NMR (600 MHz, DMSO-$d_6$) δ 8.40 (s, 1H), 7.93 (d, *J* = 7.0 Hz, 1H), 7.72 (d, *J* = 7.9 Hz, 1H), 7.68 (t, *J* = 7.6 Hz, 1H), 7.42 (d, *J* = 8.4 Hz, 1H), 7.38 (t, *J* = 7.6 Hz, 1H), 7.08 (d, *J* = 6.9 Hz, 1H), 6.12 (s, 1H), 5.43 (s, 2H), 4.96 (t, *J* = 6 Hz, 2H), 4.89 (t, *J* = 6 Hz, 2H), 2.73 (q, *J* = 7.8 Hz, 2H), 1.10 (t, *J* = 7.4 Hz, 3H). NMR (150 MHz, DMSO-$d_6$) δ 165.7, 163.8, 160.5, 151.1, 142.8, 145.1, 142.7, 146.2, 141.5, 131.9, 128.4, 110.3, 113.5, 113.8, 111.9, 101.7, |

| | | |
|---|---|---|
| | | 63.4, 52.1, 50.4, 45.1, 20.6. HRMS (ESI) calcd for $C_{21}H_{21}N_4O_5$ $[M+H]^+$: 409.1612; found: 409.1607. |
| CD11 | 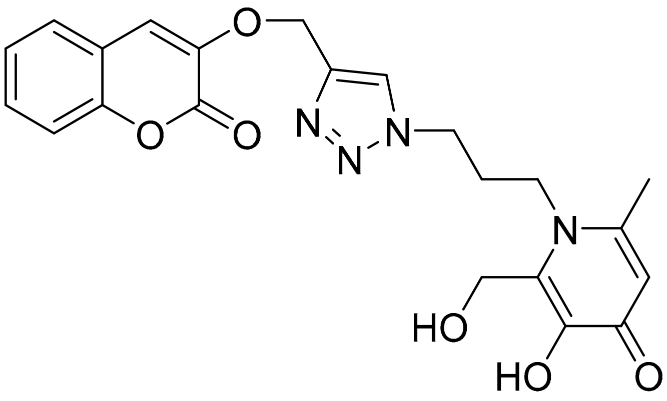 | **2-ethyl-3-hydroxy-1-(3-(4-(((2-oxo-2H-chromen-3-yl)oxy)methyl)-1H-1,2,3-triazol-1-yl)propyl)pyridin-4(*1H*)-one:** white solid, yield: 92%; m.p.: 195-198 ºC; $^1$H NMR (600 MHz, DMSO-*d*6) δ 8.49 (s, 1H), 7.76 (d, *J* = 8.0 Hz, 1H), 7.67 (t, *J* = 7.2 Hz, 1H), 7.42 (d, *J* = 7.2 Hz, 1H), 7.35 (t, *J* = 7.8 Hz, 1H), 7.11 (s, 1H), 6.17 (s, 1H), 5.46 (s, 2H), 4.76 (s, 2H), 4.63 (t, *J* = 6.6 Hz, 2H), 2.56 (s, 3H), 2.55 (s, 2H), 2.39-2.45 (m, 2H). $^{13}$C NMR (150 MHz, DMSO-*d*6) δ 166.4, 165.7, 160.3, 154.2, 140.8, 143.6, 142.7, 141.9, 141.2, 130.7, 128.5, 122.5, 117.1, 116.5, 112.3, 103.7, 63.3, 51.5, 50.2, 46.8, 31.2. 19.2. HRMS (ESI) calcd for $C_{22}H_{23}N_4O_6$ $[M+H]^+$: 439.1616; found: 439.1603. |
| CD12 | 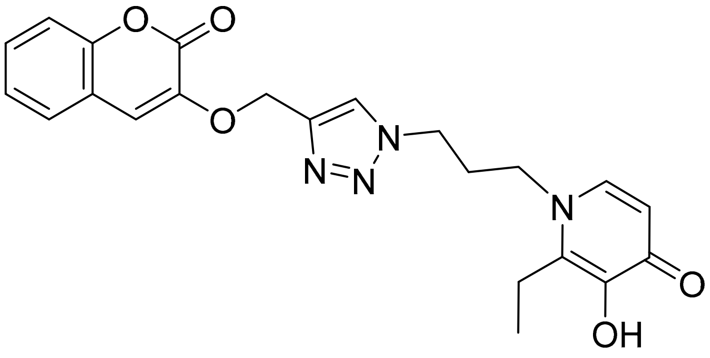 | **3-hydroxy-2-(hydroxymethyl)-6-methyl-1-(2-(4-(((2-oxo-2H-chromen-3-yl)oxy)methyl)-1H-1,2,3-triazol-1-yl)ethyl)pyridin-4(*1H*)-one:** white solid, yield: 85%; m.p.: 168-170 ºC; $^1$H NMR (600 MHz, DMSO-*d*6) δ 8.40 (s, 1H), 7.93 (d, *J* = 7.0 Hz, 1H), 7.72 (d, *J* = 7.9 Hz, 1H), 7.68 (t, *J* = 7.6 Hz, 1H), 7.42 (d, *J* = 8.4 Hz, 1H), 7.38 (t, *J* = 7.6 Hz, 1H), 7.08 (d, *J* = 6.9 Hz, 1H), 6.12 (s, 1H), 5.43 (s, 2H), 4.96 (t, *J* = 6 Hz, 2H), 4.89 (t, *J* = 6 Hz, 2H), 2.73 (q, *J* = 7.8 Hz, 2H), 1.10 (t, *J* = 7.4 Hz, |

| | | |
|---|---|---|
| | | 3H). NMR (150 MHz, DMSO-$d_6$) δ 165.7, 163.8, 160.5, 151.1, 142.8, 145.1, 142.7, 146.2, 141.5, 131.9, 128.4, 110.3, 113.5, 113.8, 111.9, 101.7, 63.4, 52.1, 50.4, 45.1, 20.6. HRMS (ESI) calcd for $C_{21}H_{21}N_4O_5$ $[M+H]^+$: 409.1612; found: 409.1607. |

**Table S3.** The tagged chemical shifts (ppm) and function groups from the indicated metabolites

| No. | HMDB No. | Metabolites Name | Tagged chemical shift (ppm) | Function groups |
|---|---|---|---|---|
| 1 | HMDB0001857 | 1,3-dimethylurate | 3.308 | $CH_3$ |
| 2 | HMDB0001860 | 1,7-dimethylxanthine | 7.830 | NH |
| 3 | HMDB0000008 | 2-hydroxybutyrate | 0.887 | $CH_3$ |
| 4 | HMDB0000694 | 2-hydroxyglutarate | 4.017 | $CH_2$ |
| 5 | HMDB0001863 | 2-hydroxyvalerate | 0.900 | $CH_3$ |
| 6 | HMDB0000208 | 2-oxoglutarate | 2.429, 2.997 | $CH_2$, $CH_2$ |
| 7 | HMDB0000357 | 3-hydroxybutyrate | 1.188 | $CH_3$ |
| 8 | HMDB0000042 | acetate | 1.908 | $CH_3$ |
| 9 | HMDB0000060 | acetoacetate | 2.272 | $CH_3$ |
| 10 | HMDB0001659 | acetone | 2.225 | $CH_3$ |
| 11 | HMDB0001341 | ADP | 8.254, 8.507 | NH |
| 12 | HMDB0000161 | alanine | 1.469 | $CH_3$ |
| 13 | HMDB0000045 | AMP | 8.247, 8.576 | NH |
| 14 | HMDB0000191 | aspartate | 2.669, 2.801 | $CH_2$ |
| 15 | HMDB0000538 | ATP | 8.245, 8.500 | NH |
| 16 | HMDB0000043 | betaine | 3.252 | $CH_3$ |
| 17 | HMDB0000039 | butyrate | 0.882 | $CH_3$ |
| 18 | HMDB0000097 | choline | 3.189 | $CH_3$ |
| 19 | HMDB0000094 | citrate | 2.526 | $CH_3$ |
| 20 | HMDB0000193 | isocitrate | 2.990 | $CH_3$ |
| 21 | HMDB0000064 | creatine | 3.025 | $CH_3$ |
| 22 | HMDB0000064 | creatine phosphate | 3.940 | $CH_3$ |
| 23 | HMDB0000562 | creatinine | 4.047 | $CH_3$ |
| 24 | HMDB0000087 | dimethylamine | 2.715 | $CH_3$ |
| 25 | HMDB0000108 | ethanol | 1.173 | $CH_3$ |
| 26 | HMDB0001887 | ethylene glycol | 3.662 | $CH_3$ |
| 27 | HMDB0000142 | formate | 8.444 | H |
| 28 | HMDB0000134 | fumarate | 6.509 | =CH |
| 29 | HMDB0000107 | galactitol | 3.688 | CH |
| 30 | HMDB0000122 | glucose | 4.637, 5.226 | CH |
| 31 | HMDB0000148 | glutamate | 2.348 | $CH_2$ |
| 32 | HMDB0000641 | glutamine | 2.445 | $CH_2$ |
| 33 | HMDB0000125 | glutathione | 2.965 | $CH_2$ |
| 34 | HMDB0000123 | glycine | 3.575 | $CH_2$ |
| 35 | HMDB0000128 | guanidoacetate | 3.785 | $CH_2$ |

| | | | | |
|---|---|---|---|---|
| 36 | HMDB0000177 | histidine | 7.082, 7.822 | =CH |
| 37 | HMDB0000157 | hypoxanthine | 8.179, 8.201 | =CH |
| 38 | HMDB0000195 | inosine | 8.224, 8.333 | =CH |
| 39 | HMDB0000172 | isoleucine | 0.926, 0.998 | $CH_3$, $CH_2$ |
| 40 | HMDB0000190 | lactate | 4.118 | CH |
| 41 | HMDB0000687 | leucine | 0.948 | $CH_3$ |
| 42 | HMDB0000182 | lysine | 1.715 | $CH_2$ |
| 43 | HMDB0000691 | malonate | 3.119 | $CH_2$ |
| 44 | HMDB0001138 | N-acetylglutamate | 2.021 | $CH_3$ |
| 45 | HMDB0006029 | N-acetylglutamine | 2.025 | $CH_3$ |
| 46 | HMDB0000532 | N-acetylglycine | 2.028 | $CH_3$ |
| 47 | HMDB0000902 | NAD+ | 9.329 | CH |
| 48 | HMDB0001406 | niacinamide | 8.929 | $NH_2$ |
| 49 | HMDB0001565 | phosphocholine | 3.206 | $CH_3$ |
| 50 | HMDB0000210 | pantothenate | 0.883, 0.918 | $CH_3$ |
| 51 | HMDB0000159 | phenylalanine | 7.318, 7.367, 7.419 | CH |
| 52 | HMDB0000243 | pyruvate | 2.363 | $CH_3$ |
| 53 | HMDB0000086 | glycero-3-phosphocholine | 3.215 | $CH_3$ |
| 54 | HMDB0000254 | succinate | 2.394 | $CH_2$ |
| 55 | HMDB0000251 | taurine | 3.417 | $CH_2$ |
| 56 | HMDB0000925 | trimethylamine-N-oxide | 3.253 | $CH_3$ |
| 57 | HMDB0000158 | tyrosine | 6.887, 7.182 | CH |
| 58 | HMDB0000883 | valine | 1.030 | $CH_3$ |

**Table S4.** Detailed statistical information for each group of differential metabolites.

| CD1 vs. Ctrl | | | | |
|---|---|---|---|---|
| | p | FC | VIP | p(corr) |
| glycine | 0.0004 | 1.2891 | 1.4341 | 0.8899 |
| guanidoacetate | 0.0131 | 1.1860 | 1.0232 | 0.6426 |
| lysine | 0.0234 | 1.1903 | 1.1271 | 0.5334 |
| aspartate | 0.0037 | -1.1800 | 1.1183 | -0.7673 |
| galactitol | 0.0481 | -1.1578 | 1.2110 | -0.6207 |
| taurine | 0.0323 | -1.1877 | 1.2788 | -0.6144 |
| CD2 vs. Ctrl | | | | |
| | p | r | VIP | p(corr) |
| galactitol | 0.0469 | 1.1739 | 1.1184 | 0.5297 |
| glutamate | 0.0264 | 1.3199 | 1.9076 | 0.7066 |
| glutamine | 0.0156 | 1.2160 | 1.4088 | 0.5259 |
| taurine | 0.0004 | -1.2242 | 1.4384 | -0.7824 |
| inosine | 0.0052 | -1.1828 | 1.1209 | -0.9053 |
| lysine | 0.0099 | -1.1788 | 1.3903 | -0.6761 |
| CD3 vs. Ctrl | | | | |
| | p | FC | VIP | p(corr) |
| phenylalanine | 0.0000 | 1.2826 | 1.3158 | 0.8671 |
| glutamate | 0.0135 | 1.3637 | 1.8608 | 0.6218 |
| glutamine | 0.0076 | 1.3618 | 2.0673 | 0.6957 |
| NAD+ | 0.0347 | 1.2587 | 1.6972 | 0.6305 |
| taurine | 0.0000 | -1.7013 | 2.9456 | -0.9636 |
| aspartate | 0.0000 | -1.1881 | 1.6817 | -0.9370 |
| lysine | 0.0080 | -1.2176 | 1.1799 | -0.6805 |
| CD4 vs. Ctrl | | | | |
| | p | FC | VIP | p(corr) |
| galactitol | 0.0000 | 1.7099 | 1.4126 | 0.9057 |
| glutamate | 0.0010 | 1.6298 | 1.3248 | 0.8152 |
| glutamine | 0.0007 | 1.9457 | 1.5801 | 0.8393 |
| glutathione | 0.0015 | 10.5329 | 2.9545 | 0.8150 |
| NAD+ | 0.0001 | 2.1130 | 1.6017 | 0.7937 |
| taurine | 0.0000 | -2.3755 | 1.8588 | -0.9813 |
| lysine | 0.0000 | -1.6173 | 1.3681 | -0.9545 |
| CD5 vs. Ctrl | | | | |
| | p | FC | VIP | p(corr) |
| glutamate | 0.0013 | 1.7729 | 1.2967 | 0.4243 |
| glutamine | 0.0011 | 2.1033 | 1.5150 | 0.9699 |
| ADP | 0.0002 | 1.4419 | 1.0858 | 0.8865 |
| taurine | 0.0000 | -2.2449 | 1.7107 | -0.9668 |
| lactate | 0.0222 | -1.9491 | 1.2174 | -0.5926 |

| | p | FC | VIP | p(corr) |
|---|---|---|---|---|
| CD6 vs. Ctrl | | | | |
| | p | FC | VIP | p(corr) |
| glycine | 0.0000 | 1.1602 | 1.2528 | 0.8705 |
| lactate | 0.0230 | 1.7565 | 2.0297 | 0.6077 |
| aspartate | 0.0000 | -1.2253 | 1.4525 | -0.9353 |
| taurine | 0.0000 | -1.3156 | 1.6988 | -0.8718 |
| glutathione | 0.0458 | -1.8059 | 2.1434 | -0.6081 |
| CD7 vs. Ctrl | | | | |
| | p | FC | VIP | p(corr) |
| glutamate | 0.0048 | 2.2818 | 1.6714 | 0.7408 |
| glutamine | 0.0017 | 2.0868 | 1.7074 | 0.8359 |
| glutathione | 0.0436 | 5.4437 | 2.7678 | 0.6284 |
| lactate | 0.0439 | 7.4065 | 2.9879 | 0.5870 |
| taurine | 0.0000 | -1.6553 | 1.4108 | -0.9416 |
| inosine | 0.0005 | -1.3202 | 1.0602 | -0.8677 |
| CD8 vs. Ctrl | | | | |
| | p | FC | VIP | p(corr) |
| phenylalanine | 0.0007 | 1.2713 | 1.0910 | 0.7036 |
| glutamate | 0.0408 | 1.2283 | 1.9236 | 0.5611 |
| lactate | 0.0380 | 2.1439 | 3.9894 | 0.5583 |
| taurine | 0.0000 | -1.3363 | 2.5242 | -0.8967 |
| aspartate | 0.0000 | -1.1715 | 1.9275 | -0.9245 |
| CD9 vs. Ctrl | | | | |
| | p | FC | VIP | p(corr) |
| galactitol | 0.0033 | 3.5407 | 1.6754 | 0.7464 |
| glutathione | 0.0000 | 26.6600 | 2.8216 | 0.9351 |
| glycine | 0.0000 | 3.0527 | 1.6592 | 0.9629 |
| guanidoacetate | 0.0000 | 2.6467 | 1.5585 | 0.9734 |
| ethanol | 0.0000 | 3.2240 | 1.7033 | 0.9809 |
| aspartate | 0.0000 | -1.6037 | 1.0783 | -0.9844 |
| taurine | 0.0000 | -2.0226 | 1.3253 | -0.9890 |
| inosine | 0.0000 | -1.8396 | 1.2343 | -0.9908 |
| glutamate | 0.0000 | -2.6561 | 1.5144 | -0.8956 |
| lactate | 0.0000 | -3.7449 | 1.7378 | -0.8381 |
| CD10 vs. Ctrl | | | | |
| | p | FC | VIP | p(corr) |
| galactitol | 0.0000 | 1.4696 | 1.4135 | 0.8900 |
| glutamate | 0.0000 | 2.5836 | 2.3099 | 0.8945 |
| glutamine | 0.0000 | 2.9352 | 2.5162 | 0.9211 |
| glutathione | 0.0000 | 35.4889 | 4.5058 | 0.9305 |
| glycine | 0.0050 | 1.3031 | 1.1898 | 0.7633 |
| guanidoacetate | 0.0003 | 1.2462 | 1.0552 | 0.8145 |
| butyrate | 0.0002 | 1.2645 | 1.1557 | 0.8751 |

| | p | FC | VIP | p(corr) |
|---|---|---|---|---|
| ethanol | 0.0001 | 1.3796 | 1.3377 | 0.9163 |
| leucine | 0.0001 | 1.2226 | 1.0031 | 0.8273 |
| NAD+ | 0.0006 | -1.6655 | 1.6371 | -0.8757 |
| CD11 vs. Ctrl | | | | |
| | p | FC | VIP | p(corr) |
| taurine | 0.0000 | 1.3117 | 2.1857 | 0.8085 |
| inosine | 0.0148 | 1.3594 | 1.0711 | 0.7328 |
| lactate | 0.0394 | -1.4575 | 3.0995 | -0.7218 |
| CD12 vs. Ctrl | | | | |
| | p | FC | VIP | p(corr) |
| phenylalanine | 0.0000 | 1.3169 | 1.3661 | 0.9218 |
| 3-hydroxybutyrate | 0.0021 | 1.3813 | 1.1157 | 0.8541 |
| isoleucine | 0.0025 | 1.2886 | 1.9347 | 0.7812 |
| lactate | 0.0214 | 1.8251 | 2.5595 | 0.6218 |
| leucine | 0.0037 | 1.4152 | 2.2915 | 0.7795 |
| lysine | 0.0401 | 1.1815 | 1.4645 | 0.5843 |
| taurine | 0.0000 | -1.5662 | 2.5460 | -0.9320 |
| aspartate | 0.0000 | -1.2643 | 1.9503 | -0.9598 |
| glutamine | 0.0428 | -1.2472 | 1.5466 | -0.5901 |

**Table S5**. The results of significantly disturbed metabolic pathways for each coumarin derivative affecting the celluar U251metabolic pathways

| | Total | Expected | Hits | Raw p | -log(p) | Holmadjust | FDR | Impact |
|---|---|---|---|---|---|---|---|---|
| CD1 vs. Ctrl | | | | | | | | |
| Aminoacyl-tRNA biosynthesis | 48 | 0.19 | 3 | $5.23 \times 10^{-4}$ | 3.28 | $4.39 \times 10^{-2}$ | $4.39 \times 10^{-2}$ | 0.10 |
| Glycine, serine and threoninemetabolism | 33 | 0.13 | 2 | $6.25 \times 10^{-3}$ | 2.20 | $5.19 \times 10^{-1}$ | $2.63 \times 10^{-1}$ | 0.23 |
| Taurine and hypotaurine metabolism | 8 | 0.03 | 1 | $3.06 \times 10^{-2}$ | 1.51 | 1.00 | $6.19 \times 10^{-1}$ | 0.33 |
| CD2 vs. Ctrl | | | | | | | | |
| D-Glutamine and D-glutamate metabolism | 6 | 0.02 | 2 | $1.86 \times 10^{-4}$ | 3.73 | $1.56 \times 10^{-2}$ | $7.82 \times 10^{-3}$ | 0.25 |
| Aminoacyl-tRNA biosynthesis | 48 | 0.19 | 3 | $5.23 \times 10^{-4}$ | 3.28 | $4.29 \times 10^{-2}$ | $1.46 \times 10^{-2}$ | 0.10 |
| Arginine biosynthesis | 14 | 0.05 | 2 | $1.11 \times 10^{-3}$ | 2.95 | $9.02 \times 10^{-2}$ | $2.34 \times 10^{-2}$ | 0.19 |
| Alanine, aspartate and glutamate metabolism | 28 | 0.11 | 2 | $4.52 \times 10^{-3}$ | 2.35 | $3.61 \times 10^{-1}$ | $7.59 \times 10^{-2}$ | 0.28 |
| Taurine and hypotaurine metabolism | 8 | 0.03 | 1 | $3.06 \times 10^{-2}$ | 1.51 | 1.00 | $3.22 \times 10^{-1}$ | 0.33 |
| CD3 vs. Ctrl | | | | | | | | |
| Aminoacyl-tRNA biosynthesis | 48 | 0.22 | 5 | $4.63 \times 10^{-7}$ | 6.33 | $3.89 \times 10^{-5}$ | $3.89 \times 10^{-5}$ | 0.17 |
| Arginine biosynthesis | 14 | 0.06 | 3 | $2.01 \times 10^{-5}$ | 4.70 | $1.67 \times 10^{-3}$ | $8.46 \times 10^{-4}$ | 0.25 |
| Alanine, aspartate and glutamate metabolism | 28 | 0.13 | 3 | $1.76 \times 10^{-4}$ | 3.75 | $1.45 \times 10^{-2}$ | $4.37 \times 10^{-3}$ | 0.47 |
| D-Glutamine and D-glutamate metabolism | 6 | 0.03 | 2 | $2.60 \times 10^{-4}$ | 3.58 | $2.11 \times 10^{-2}$ | $4.37 \times 10^{-3}$ | 0.50 |
| Phenylalanine, tyrosine and tryptophan biosynthesis | 4 | 0.02 | 1 | $1.80 \times 10^{-2}$ | 1.75 | 1.00 | $1.68 \times 10^{-1}$ | 0.50 |
| CD4 vs. Ctrl | | | | | | | | |
| D-Glutamine and D-glutamate metabolism | 6 | 0.03 | 2 | $2.60 \times 10^{-4}$ | 3.58 | $2.19 \times 10^{-2}$ | $1.09 \times 10^{-2}$ | 0.50 |
| Aminoacyl-tRNA biosynthesis | 48 | 0.22 | 3 | $8.95 \times 10^{-4}$ | 3.05 | $7.34 \times 10^{-2}$ | $2.51 \times 10^{-2}$ | 0.10 |
| Arginine biosynthesis | 14 | 0.06 | 2 | $1.55 \times 10^{-3}$ | 2.81 | $1.26 \times 10^{-1}$ | $3.26 \times 10^{-2}$ | 0.19 |
| Alanine, aspartate and glutamate metabolism | 28 | 0.13 | 2 | $6.25 \times 10^{-3}$ | 2.20 | $5.00 \times 10^{-1}$ | $8.75 \times 10^{-2}$ | 0.28 |

| | | | | | | | | |
|---|---|---|---|---|---|---|---|---|
| Glutathione metabolism | 28 | 0.13 | 2 | $6.25 \times 10^{-3}$ | 2.20 | $5.00 \times 10^{-1}$ | $8.75 \times 10^{-2}$ | 0.19 |
| Taurine and hypotaurine metabolism | 8 | 0.04 | 1 | $3.56 \times 10^{-2}$ | 1.45 | 1.00 | $3.74 \times 10^{-1}$ | 0.33 |
| CD5 vs. Ctrl | | | | | | | | |
| D-Glutamine and D-glutamate metabolism | 6 | 0.02 | 2 | $1.24 \times 10^{-4}$ | 3.91 | $1.04 \times 10^{-2}$ | $5.22 \times 10^{-3}$ | 0.50 |
| Arginine biosynthesis | 14 | 0.05 | 2 | $7.46 \times 10^{-4}$ | 3.13 | $6.12 \times 10^{-2}$ | $2.09 \times 10^{-2}$ | 0.19 |
| Alanine, aspartate and glutamate metabolism | 28 | 0.09 | 2 | $3.04 \times 10^{-3}$ | 2.52 | $2.47 \times 10^{-1}$ | $6.39 \times 10^{-2}$ | 0.28 |
| Taurine and hypotaurine metabolism | 8 | 0.03 | 1 | $2.56 \times 10^{-2}$ | 1.59 | 1.00 | $2.69 \times 10^{-1}$ | 0.33 |
| CD6 vs. Ctrl | | | | | | | | |
| Glutathione metabolism | 28 | 0.09 | 2 | $3.04 \times 10^{-3}$ | 2.52 | $2.56 \times 10^{-1}$ | $2.48 \times 10^{-1}$ | 0.19 |
| Aminoacyl-tRNA biosynthesis | 48 | 0.15 | 2 | $8.85 \times 10^{-3}$ | 2.05 | $7.26 \times 10^{-1}$ | $2.48 \times 10^{-1}$ | 0.07 |
| Taurine and hypotaurine metabolism | 8 | 0.03 | 1 | $2.56 \times 10^{-2}$ | 1.59 | 1.00 | $5.37 \times 10^{-1}$ | 0.33 |
| CD7 vs. Ctrl | | | | | | | | |
| D-Glutamine and D-glutamate metabolism | 6 | 0.02 | 2 | $1.86 \times 10^{-4}$ | 3.73 | $1.56 \times 10^{-2}$ | $1.56 \times 10^{-2}$ | 0.5 |
| Arginine biosynthesis | 14 | 0.05 | 2 | $1.11 \times 10^{-3}$ | 2.95 | $9.12 \times 10^{-2}$ | $9.12 \times 10^{-2}$ | 0.19 |
| Alanine, aspartate and glutamate metabolism | 28 | 0.11 | 2 | $4.52 \times 10^{-3}$ | 2.35 | $7.59 \times 10^{-2}$ | $7.59 \times 10^{-2}$ | 0.28 |
| Glutathione metabolism | 28 | 0.11 | 2 | $4.52 \times 10^{-3}$ | 2.35 | $7.59 \times 10^{-2}$ | $7.59 \times 10^{-2}$ | 0.19 |
| Taurine and hypotaurine metabolism | 8 | 0.03 | 1 | $3.06 \times 10^{-2}$ | 1.51 | $2.86 \times 10^{-1}$ | $2.86 \times 10^{-1}$ | 0.33 |
| CD8 vs. Ctrl | | | | | | | | |
| Arginine biosynthesis | 14 | 0.05 | 2 | $7.46 \times 10^{-4}$ | 3.13 | $6.19 \times 10^{-2}$ | $2.75 \times 10^{-2}$ | 0.19 |
| Alanine, aspartate and glutamate metabolism | 28 | 0.05 | 2 | $3.04 \times 10^{-3}$ | 2.52 | $2.47 \times 10^{-2}$ | $6.39 \times 10^{-2}$ | 0.31 |
| Phenylalanine, tyrosine and tryptophan biosynthesis | 4 | 0.01 | 1 | $1.29 \times 10^{-2}$ | 1.89 | 1.00 | $2.16 \times 10^{-1}$ | 0.50 |
| CD9 vs. Ctrl | | | | | | | | |
| Glutathione metabolism | 28 | 0.18 | 3 | $5.83 \times 10^{-4}$ | 3.23 | $4.90 \times 10^{-2}$ | $4.90 \times 10^{-2}$ | 0.22 |
| Aminoacyl-tRNA biosynthesis | 48 | 0.31 | 3 | $2.87 \times 10^{-3}$ | 2.54 | $2.38 \times 10^{-1}$ | $9.00 \times 10^{-2}$ | 0.10 |

| | | | | | | | | |
|---|---|---|---|---|---|---|---|---|
| Arginine biosynthesis | 14 | 0.09 | 2 | $3.27 \times 10^{-3}$ | 2.49 | $2.68 \times 10^{-1}$ | $9.00 \times 10^{-2}$ | 0.19 |
| Alanine, aspartate and glutamate metabolism | 28 | 0.18 | 2 | $1.30 \times 10^{-2}$ | 1.89 | 1.00 | $1.66 \times 10^{-1}$ | 0.31 |
| Glycine, serine and threonine metabolism | 33 | 0.21 | 2 | $1.78 \times 10^{-2}$ | 1.75 | 1.00 | $1.66 \times 10^{-1}$ | 0.23 |
| Taurine and hypotaurine metabolism | 8 | 0.05 | 1 | $5.06 \times 10^{-2}$ | 1.30 | 1.00 | $3.03 \times 10^{-1}$ | 0.33 |
| CD10 vs. Ctrl | | | | | | | | |
| Aminoacyl-tRNA biosynthesis | 48 | 0.31 | 4 | $1.49 \times 10^{-4}$ | 3.83 | $1.25 \times 10^{-2}$ | $1.22 \times 10^{-2}$ | 0.14 |
| D-Glutamine and D-glutamate metabolism | 6 | 0.04 | 2 | $5.55 \times 10^{-4}$ | 3.26 | $4.06 \times 10^{-2}$ | $1.22 \times 10^{-2}$ | 0.50 |
| Glutathione metabolism | 28 | 0.18 | 3 | $5.83 \times 10^{-4}$ | 3.23 | $4.72 \times 10^{-2}$ | $1.22 \times 10^{-2}$ | 0.22 |
| Arginine biosynthesis | 14 | 0.09 | 2 | $3.27 \times 10^{-3}$ | 2.49 | $2.59 \times 10^{-1}$ | $4.52 \times 10^{-2}$ | 0.19 |
| Alanine, aspartate and glutamate metabolism | 28 | 0.18 | 2 | $1.30 \times 10^{-2}$ | 1.89 | $9.97 \times 10^{-1}$ | $1.36 \times 10^{-1}$ | 0.28 |
| Glycine, serine and threonine metabolism | 33 | 0.21 | 2 | $1.78 \times 10^{-2}$ | 1.75 | 1.00 | 1.49 x 10-1 | 0.23 |
| Valine, leucine and isoleucine biosynthesis | 8 | 0.05 | 1 | $5.06 \times 10^{-2}$ | 1.30 | 1.00 | $3.54 \times 10^{-1}$ | 0.25 |
| CD11 vs. Ctrl | | | | | | | | |
| Taurine and hypotaurine metabolism | 8 | 0.02 | 1 | $1.54 \times 10^{-2}$ | 1.81 | 1.00 | 1.00 | 0.33 |
| CD12 vs. Ctrl | | | | | | | | |
| Aminoacyl-tRNA biosynthesis | 48 | 0.28 | 6 | $5.03 \times 10^{-8}$ | 7.30 | $4.23 \times 10^{-6}$ | $4.23 \times 10^{-6}$ | 0.21 |
| Valine, leucine and isoleucine biosynthesis | 8 | 0.05 | 2 | $8.25 \times 10^{-4}$ | 3.08 | $6.84 \times 10^{-2}$ | $3.46 \times 10^{-2}$ | 0.25 |
| Alanine, aspartate and glutamate metabolism | 28 | 0.16 | 2 | $1.05 \times 10^{-2}$ | 1.98 | $8.49 \times 10^{-1}$ | $2.20 \times 10^{-1}$ | 0.34 |
| Phenylalanine, tyrosine and tryptophan biosynthesis | 4 | 0.02 | 1 | $2.30 \times 10^{-2}$ | 1.64 | 1.00 | $3.21 \times 10^{-1}$ | 0.50 |
